# Severe Chronic Tubulointerstitial Nephropathy in a Geriatric Pet Chinchilla (*Chinchilla lanigera*): A Case Report and Literature Review

**DOI:** 10.3390/ani16213451

**Published:** 2026-11-01

**Authors:** Mihaela Niculae, Smaranda Crăciun, Raluca Marica, Maria Carmen Turcu, Lucia Victoria Bel, Andrei Răzvan Codea, Raul Alexandru Pop, Simon L. Priestnall

**Affiliations:** 1 Department of Infectious Diseases, Faculty of Veterinary Medicine, University of Agricultural Sciences and Veterinary Medicine Cluj-Napoca, 400372 Cluj-Napoca, Romania; mihaela.niculae@usamvcluj.ro; 2 Department of Microbiology, Immunology and Epidemiology, Faculty of Veterinary Medicine, University of Agricultural Sciences and Veterinary Medicine Cluj-Napoca, 400372 Cluj-Napoca, Romania; smaranda.craciun@usamvcluj.ro; 3 Department of Pathology, Faculty of Veterinary Medicine, University of Agricultural Sciences and Veterinary Medicine Cluj-Napoca, 400372 Cluj-Napoca, Romania; 4 New Companion Animals Clinic, Department of Surgery and Intensive Care, Faculty of Veterinary Medicine, University of Agricultural Sciences and Veterinary Medicine Cluj-Napoca, 400372 Cluj-Napoca, Romania; lucia.bel@usamvcluj.ro; 5 Department of Internal Medicine, Faculty of Veterinary Medicine, University of Agricultural Sciences and Veterinary Medicine Cluj-Napoca, 400372 Cluj-Napoca, Romania; razvan.codea@usamvcluj.ro; 6 Department of Obstetrics and Gynecology, Faculty of Veterinary Medicine, University of Agricultural Science and Veterinary Medicine Cluj-Napoca, 400372 Cluj-Napoca, Romania; alexandru.pop@usamvcluj.ro; 7 Department of Pathobiology and Population Sciences, The Royal Veterinary College, Hertfordshire AL9 7TA, UK; spriestnall@rvc.ac.uk

**Keywords:** *Chinchilla lanigera*, chronic tubulointerstitial nephropathy, renal mineralization, *Klebsiella pneumoniae*, multidrug resistance, pulmonary congestion, heat stress, post-mortem examination

## Abstract

Chronic renal disease is recognized in geriatric companion mammals, but detailed clinicopathological descriptions in chinchillas remain limited. An 11-year-old neutered female pet chinchilla (*Chinchilla lanigera*) with a six-month history of progressive weight loss and hyporexia developed acute clinical deterioration following approximately 8 h of indoor heat exposure (estimated maximum 29 °C). Anorexia, lethargy, absence of fecal output, and progressive dyspnoea preceded death. Necropsy revealed severe bilateral chronic renal lesions, gastrointestinal gaseous distension, and severe pulmonary congestion. Histopathology documented chronic tubulointerstitial nephropathy with interstitial fibrosis supported by Masson–Goldner trichrome staining and mineral deposits demonstrated by von Kossa staining. Periodic acid–Schiff staining highlighted glomerular and tubular basement membranes. Marked pulmonary vascular congestion was confirmed histologically, whereas pulmonary oedema and established pneumonia were not demonstrated in the examined sections. Together with the progressive terminal dyspnoea, these findings indicated clinically important respiratory abnormalities, although their underlying mechanism and contribution to death remained uncertain. Pure cultures of *Klebsiella pneumoniae* were recovered from six anatomical sites, with all isolates exhibiting an identical antimicrobial susceptibility profile and a reported multidrug-resistant phenotype. No bacterial-associated inflammatory lesions were identified in the histologically examined tissues, providing no morphological evidence of established disseminated septic disease. Given the uncertain time of death and a possible post-mortem interval of up to approximately 6 h, ante-mortem infection could not be reliably distinguished from perimortem dissemination or post-mortem translocation. Environmental heat exposure may have contributed to acute deterioration in an animal with severe chronic disease, although heat stroke could not be established retrospectively. This case supports a multifactorial interpretation and highlights the potential One Health relevance of antimicrobial resistance in companion animals, without demonstrating transmission.

## Simple Summary

Chinchillas can live well beyond 10 years, but relatively little is published about diseases in older pets. We investigated the death of an 11-year-old female chinchilla with progressive weight loss and reduced appetite, followed by rapid deterioration after environmental heat exposure. Necropsy revealed severe chronic renal disease, gastrointestinal gaseous distension, and severe pulmonary congestion. Additional renal histochemical stains supported fibrosis, basement-membrane thickening, and mineral deposition. Histology confirmed marked pulmonary vascular congestion but not pulmonary oedema or pneumonia; this finding may have been agonal and cannot independently establish respiratory failure. Pure cultures of multidrug-resistant *Klebsiella pneumoniae* with an identical in vitro antimicrobial susceptibility profile were isolated from several organs; however, histopathology did not identify bacterial-associated inflammatory lesions in the tissues examined. Their contribution to death and any zoonotic significance remains unproven. Heat exposure may have precipitated decompensation in an animal with severe chronic disease, although heat stroke was not confirmed. This case highlights the value of combined pathology and microbiology, and the need for cautious interpretation of resistant bacterial isolates from pets.

## 1. Introduction

The long-tailed chinchilla (*Chinchilla lanigera*) is an herbivorous hystricomorph rodent with a comparatively long lifespan. Pet chinchillas commonly live beyond 10 years, and longevity of approximately 17 years has been documented [1,2]. As more animals reach advanced age, standardized husbandry, preventive health monitoring, and species-specific veterinary care become increasingly important [3]. Maintaining health throughout this extended lifespan requires adequate nutrition, including a high-fiber diet, suitable housing, environmental enrichment and regular exercise, as well as appropriate environmental conditions. Among these, temperature control is particularly relevant in chinchillas because, as a species adapted to cool environments, they have high thermal insulation associated with their exceptionally dense fur and a limited capacity for heat dissipation. Experimental studies in *C. lanigera* have demonstrated an increased risk of hyperthermia at high ambient temperatures around 30 °C [4]. Accordingly, appropriate control of ambient temperature and ventilation represents an important component of captive chinchilla husbandry [5,6].

Veterinary care for companion chinchillas has advanced substantially, with broader recognition and treatment of dental, gastrointestinal, orthopedic, cardiovascular, reproductive, and neoplastic disorders [7,8,9,10,11,12]. Individual reports have described bacterial glomerulonephritis [13], renal and myocardial lymphoma [14], atrial septal defect [15], granulosa cell tumor with pyometra [16], metastatic uterine lymphoma [17], diaphragmatic hernia [18], cleft palate [19], long-bone fracture repair [20], obstructive gastrointestinal disease [21], and regional anesthesia techniques [22].

Bacterial infections remain clinically relevant in laboratory, farmed, and companion chinchillas. Reported pathogens include *Pseudomonas aeruginosa* [23], *Salmonella enterica serovar* Typhimurium [24], *Streptococcus equi subsp. zooepidemicus* [25,26], and *Streptococcus canis* [27]. In addition, a large retrospective necropsy survey in farmed chinchillas identified *Escherichia coli*, *S. enterica* (serovars Typhimurium, Arizonae and Enteritidis), *P. aeruginosa*, *Yersinia pseudotuberculosis*, *Yersinia enterocolitica*, *Klebsiella pneumoniae*, and *Clostridium perfringens* as the principal bacterial agents associated with fatal enteric disease, whereas *Pasteurella multocida*, *Bordetella bronchiseptica*, *P. aeruginosa*, *Streptococcus* spp., *K. pneumoniae*, and *Listeria monocytogenes* were among the pathogens most frequently recovered from cases of pneumonia [10]. As a One Health pathogen, *K. pneumoniae* is of particular concern when antimicrobial resistance, including reduced carbapenem susceptibility, is reported in companion animals. Shared or closely related *K. pneumoniae* and resistant Enterobacterales have been described in cohabiting animals and people, although detecting the same species in a pet does not prove owner exposure or zoonotic transmission [28,29].

Despite the expanding literature, comprehensive case reports integrating clinical history, necropsy, histopathology, microbiology, and antimicrobial susceptibility testing remain uncommon in geriatric companion chinchillas. In particular, the pathological significance of multiorgan recovery of opportunistic bacteria can be difficult to determine when severe chronic disease and terminal physiological stress are present.

The present report describes an 11-year-old companion chinchilla with severe chronic renal lesions, pulmonary and gastrointestinal abnormalities, and multiorgan isolation of multidrug-resistant *K. pneumoniae*. The aims were to characterize the main gross lesions, evaluate the significance of the microbiological findings in the context of severe chronic disease, and assess the potential contribution of environmental heat exposure to the terminal clinical deterioration. The renal findings are also compared with previously reported renal lesions and renal-associated disorders in chinchillas.

## 2. Detailed Case Presentation

### 2.1. History

The body of an 11-year-old neutered female pet chinchilla (*Chinchilla lanigera*) was submitted to the Department of Pathology, Faculty of Veterinary Medicine, Cluj-Napoca, Romania, for post-mortem examination. The medical history included surgical repair of a left forelimb fracture at six months of age and elective ovariohysterectomy at two years of age; recovery from both procedures was reportedly uneventful. Short perioperative courses of enrofloxacin were administered for these two procedures, with no further systemic antimicrobial therapy administered throughout the remainder of the animal’s life.

According to the owner, progressive body-weight loss and gradually reduced appetite had been evident for approximately six months. The animal remained relatively active during most of this period and had no reported respiratory, digestive, neurological, or behavioral abnormalities. Approximately 48 h before death, the chinchilla was inadvertently exposed for approximately 8 h to an elevated indoor ambient temperature, estimated to have reached a maximum of 29 °C. The cage remained in the shade, and the animal had ad libitum access to drinking water; however, the room had no active ventilation or air conditioning. Acute clinical deterioration occurred following this period of environmental heat exposure. The animal developed lethargy, complete anorexia, absence of fecal output, and progressive dyspnoea during the final 24 h. The owner initiated assisted syringe-feeding with a commercial herbivore recovery diet (Critical Care^®^, Oxbow Animal Health, Omaha, NE, USA), but the animal died within 24 h of the onset of severe signs. No ante-mortem core body temperature, hematology, serum biochemistry, urinalysis, blood-pressure measurement, or diagnostic imaging were performed.

The animal was last observed alive at approximately 03:30 and was found dead at approximately 09:00. Post-mortem examination was initiated within 30 min of discovery; accordingly, the estimated death-to-necropsy interval could have ranged from approximately 30 min to 6 h, depending on the unknown time of death. The temperature at which the body was held before necropsy was not recorded in the available case documentation. Organ specimens were subsequently refrigerated and delivered to the microbiology laboratory approximately 1 h after sampling. This uncertainty was considered when interpreting tissue cultures and gastrointestinal gas accumulation.

### 2.2. Gross Pathology

Post-mortem examination revealed diffuse gaseous distension of the stomach, cecum, and large intestine, with multiple markedly dilated bowel loops containing only small amounts of dry ingesta. No gross evidence of intestinal obstruction, perforation, fibrinous peritonitis, or diffuse haemorrhage was identified. In the absence of a gross obstructive lesion, gastrointestinal hypomotility was considered a possible contributor to the observed gaseous distension; however, terminal or post-mortem gas accumulation could not be excluded.

**Figure 1:**
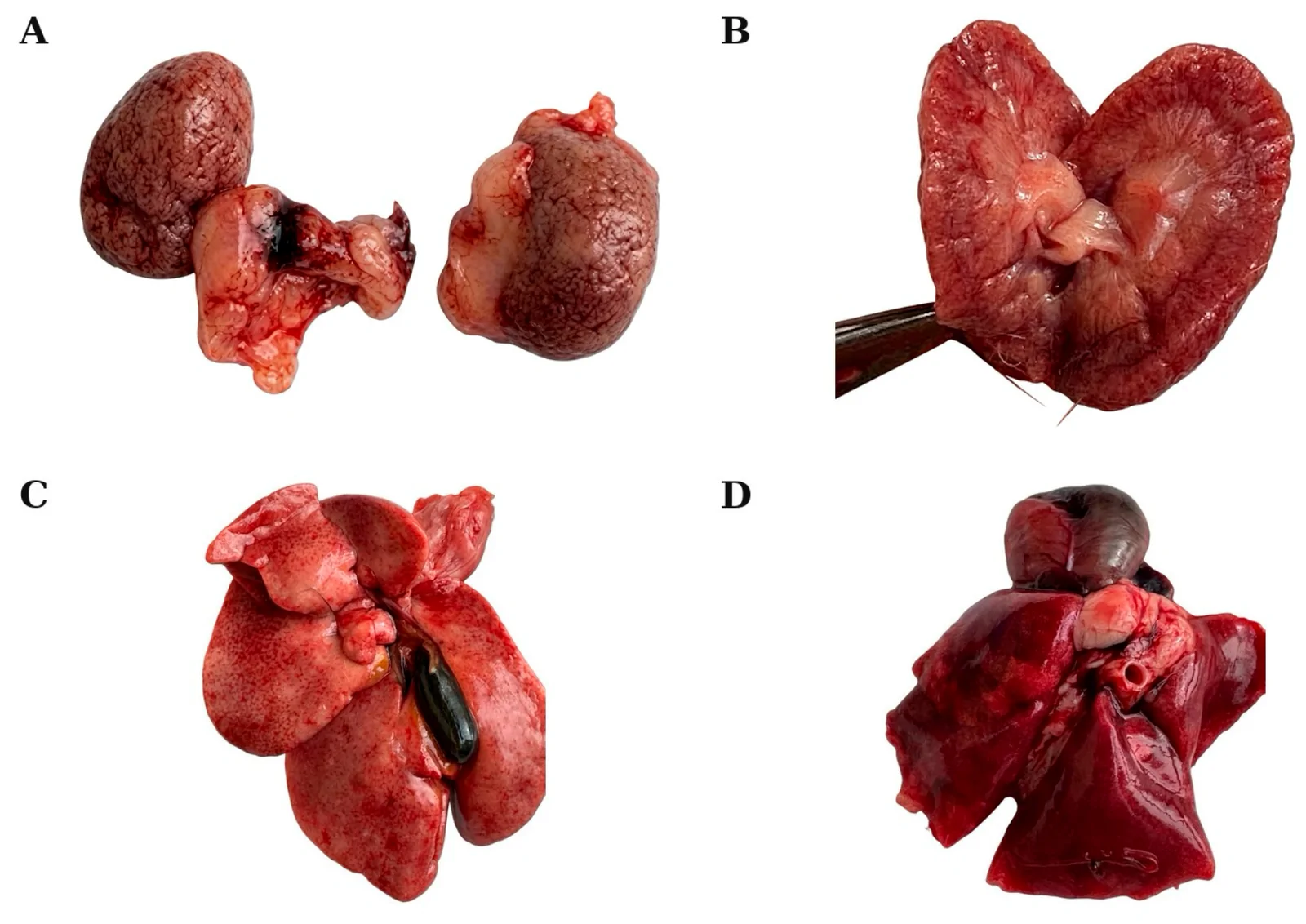
Gross necropsy findings in an 11-year-old companion chinchilla. (**A**) Markedly irregular external contours of both kidneys with fine-to-coarse nodularity, multifocal cortical depressions, diffuse cortical wrinkling, multifocal pale-gray discoloration, and dark-red congestion. (**B**) Cut surface of the kidney showing chronic architectural distortion and reduced corticomedullary distinction. (**C**) Liver showing mottled pale discoloration, accentuated lobular pattern, and a moderately distended dark-green gallbladder. (**D**) The lungs presented severe diffuse bilateral dark-red pulmonary congestion; these organs appear expanded and poorly collapsed, with a gross appearance suggestive of concurrent pulmonary oedema. The enlarged, rounded cardiac silhouette is also visible in this panel.

Both kidneys had markedly irregular external contours with fine-to-coarse nodularity, multifocal cortical depressions, diffuse cortical wrinkling, multifocal pale-gray discoloration, and dark-red congestion (Figure 1A). On cut surface, corticomedullary distinction was reduced (Figure 1B). One kidney contained a well-demarcated, indented cortical lesion grossly suggestive of a chronic infarct.

The liver had multifocal-to-coalescing pale discoloration with accentuation of the lobular pattern. The capsular surface was smooth, without other visible changes. The gallbladder was moderately distended with dark-green bile (Figure 1C).

Thoracic examination revealed severe diffuse bilateral dark-red pulmonary congestion involving all lung lobes. The lungs were expanded and poorly collapsed, and their gross appearance was considered suggestive of concurrent pulmonary oedema (Figure 1D). No gross pulmonary abscesses, fibrinous pleuritis, or focal consolidations were identified.

### 2.3. Histopathology

Samples of liver, kidneys and lungs were fixed in 10% neutral-buffered formalin for at least 48 h, embedded in paraffin, sectioned at 2–4 µm, and stained with hematoxylin and eosin for routine histopathological examination [30]. Additional sections of renal tissue were stained with Masson–Goldner trichrome to evaluate interstitial collagen deposition, periodic acid–Schiff (PAS) to assess renal basement membranes, and von Kossa to characterize mineral deposits. Sections were examined with an Olympus BX-40 light microscope, and representative photomicrographs were acquired with an Olympus SC180 digital camera and cellSens software (Olympus Corporation, Tokyo, Japan).

**Figure 2:**
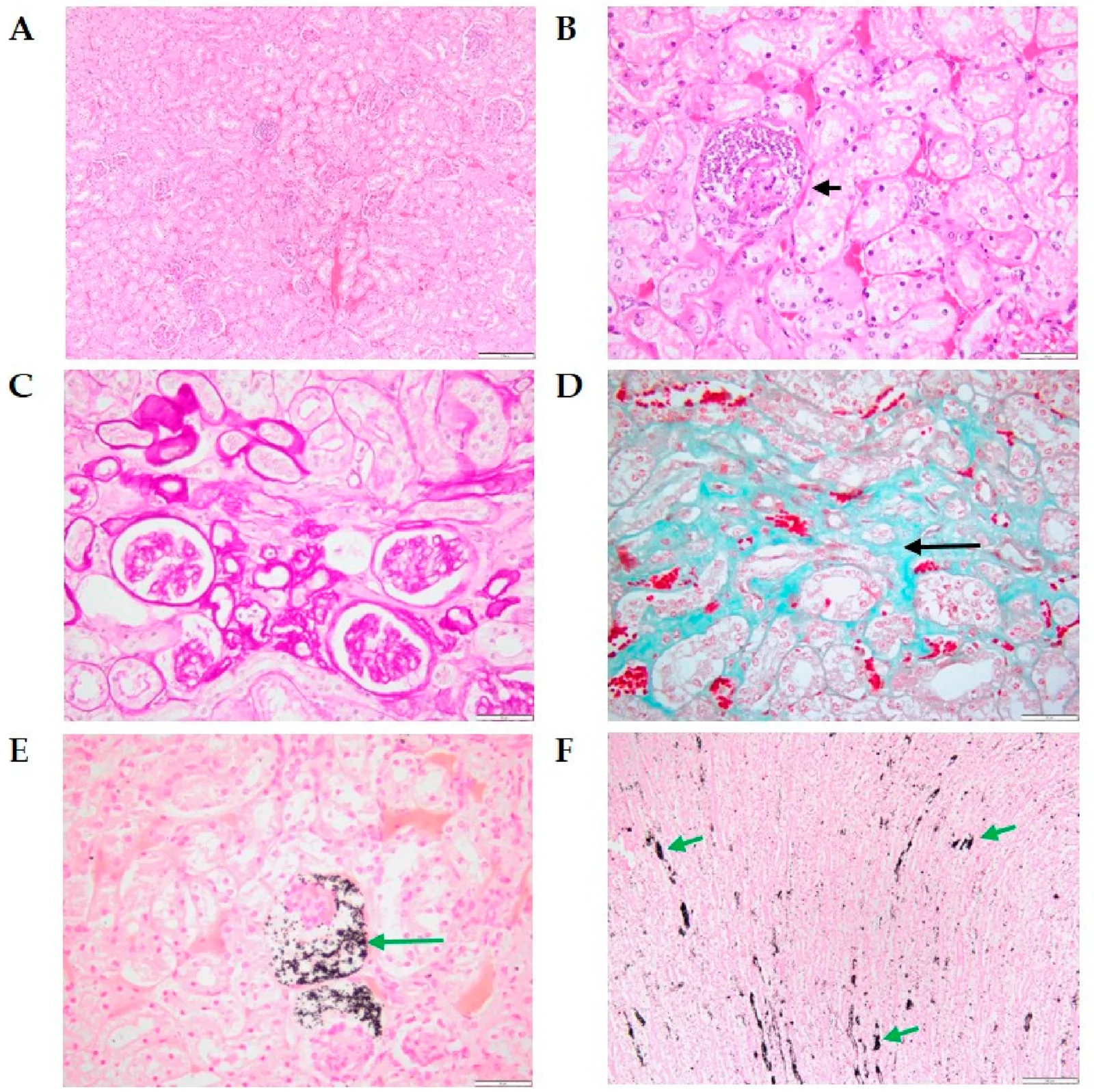
Histological and histochemical findings in the kidneys of an 11-year-old companion chinchilla. (**A**) Multifocal tubular degeneration and atrophy with interstitial expansion and congestion (H&E). (**B**) Glomerular mineralization (arrow) and adjacent tubular degeneration and atrophy (H&E). (**C**) PAS staining highlighting glomerular and tubular basement membranes. (**D**) Interstitial collagen deposition supporting renal interstitial fibrosis (Masson–Goldner trichrome). (**E**) von Kossa -positive mineral deposits associated with glomerular structures. (**F**) Multifocal medullary mineralization (von Kossa). Scale bars = 200 µm (**A**,**F**) and 50 µm (**B**–**E**).

Histological examination of both kidneys revealed marked architectural alterations consistent with advanced chronic renal injury. Multifocally, glomeruli were distended by abundant cellular and nuclear debris and contained deeply basophilic mineral deposits (Figure 2B). Renal tubules exhibited degenerative and atrophic changes, including reduced epithelial cell size, pale eosinophilic cytoplasm, and small hyperchromatic nuclei. The affected tubules were separated by expanded interstitial tissue containing pale eosinophilic fibrillar material (Figure 2A,B). PAS staining pointed out thickening of the glomerular and tubular basement membranes (Figure 2C), while Masson–Goldner trichrome staining highlighted interstitial collagen deposition, supporting the presence of renal interstitial fibrosis (Figure 2D). von Kossa staining confirmed calcium deposition in the medullary interstitium and glomerular space (Figure 2E,F). No intralesional bacterial colonies or foci of suppurative or embolic nephritis were identified in the examined sections. The overall morphological diagnosis was severe bilateral chronic tubulointerstitial nephropathy with glomerular and medullary mineralization.

**Figure 3:**
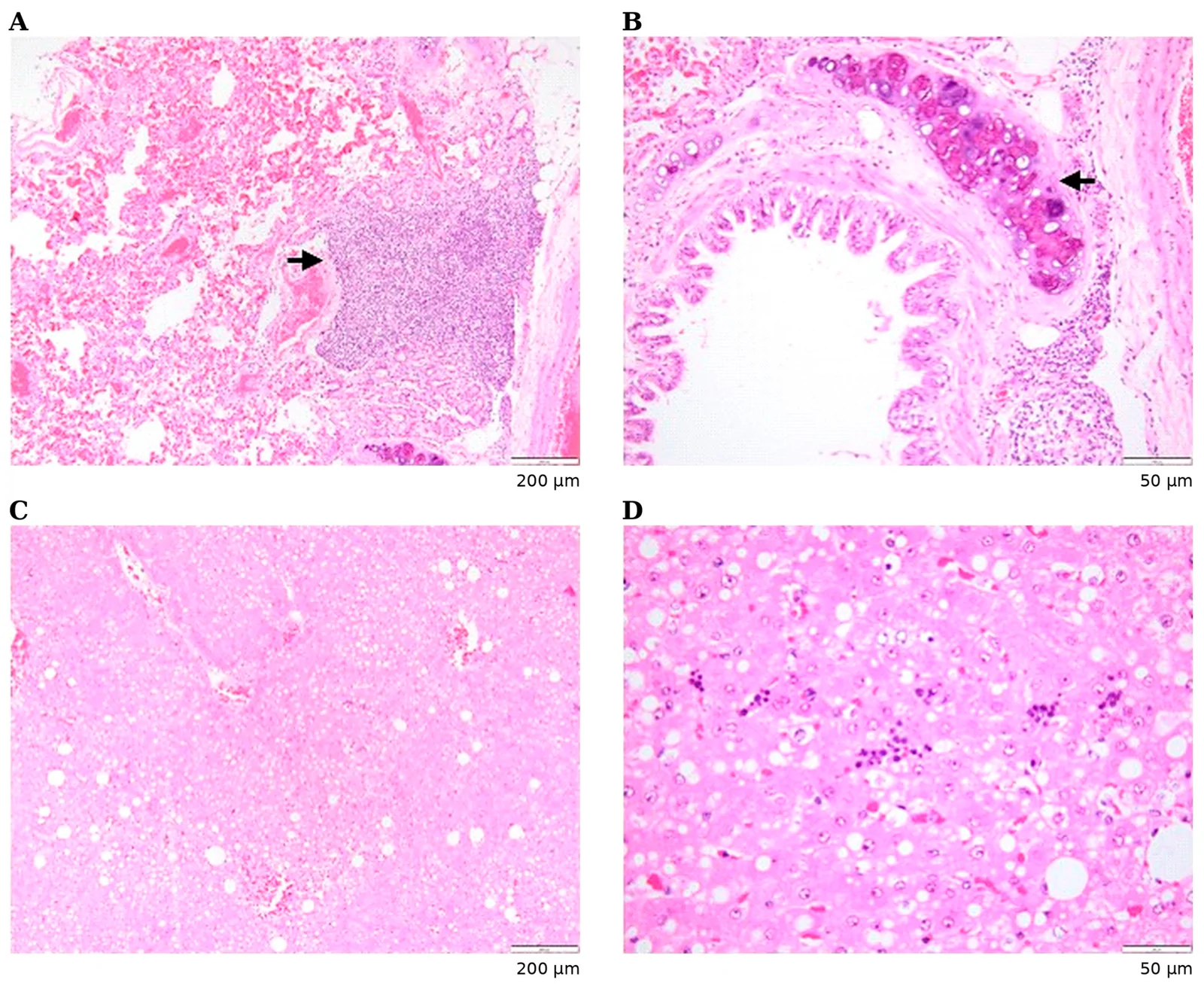
Histological findings in the lung and liver of an 11-year-old companion chinchilla. (**A**) Lung: marked diffuse vascular congestion with bronchus-associated lymphoid tissue (arrow). (**B**) Lung: bronchial cartilage within expected morphological limits and mildly prominent peribronchial smooth muscle, a borderline finding of uncertain pathological significance. (**C**) Liver: mild diffuse hepatocellular vacuolar change. (**D**) Liver: multifocal extramedullary haematopoiesis. H&E stain; scale bars = 200 µm (**A**,**C**) and 50 µm (**B**,**D**).

Pulmonary sections showed marked diffuse vascular congestion (Figure 3A). Multifocal bronchus-associated lymphoid tissue (BALT) was present and was considered a background finding. No significant alveolar accumulation of oedematous or proteinaceous material, fibrin deposition, alveolar haemorrhage, fibrin microthrombi, or other evidence of acute alveolar injury was identified in the examined sections. No established suppurative bronchopneumonia or intralesional bacterial colonies were observed. Bronchial cartilage was within expected morphological limits, while the peribronchial smooth muscle was mildly prominent, a borderline finding of uncertain pathological significance (Figure 3B).

The liver showed mild diffuse hepatocellular vacuolar change and multifocal extramedullary haematopoiesis with occasional megakaryocytes (Figure 3C,D). Hepatocellular anisocytosis, karyomegaly, and multinucleation were present and were considered background findings in this geriatric rodent. No significant suppurative, necrotizing, or bacteria-associated hepatitis was identified in the examined sections.

### 2.4. Microbiological Examination and In Vitro Antimicrobial Susceptibility Testing

Organ samples collected at post-mortem examination were placed individually into sterile containers and processed immediately by the Department of Microbiology. To minimize surface contamination, the external surface of each organ was seared with a heated sterile blade, after which a fresh incision was made using a sterile scalpel and a sterile swab was inserted into the internal parenchyma and subsequently inoculated onto Columbia agar supplemented with 10% sheep blood (BioMaxima S.A., Lublin, Poland) and MacConkey agar (Merck, Darmstadt, Germany), following the laboratory’s routine diagnostic workflow [31], and incubated aerobically at 37 °C for 24–48 h.

Representative colonies were subcultured to purity and identified by matrix-assisted laser desorption/ionization time-of-flight mass spectrometry using the MALDI Biotyper^®^ Sirius System (Bruker, Ettlingen, Germany), according to the manufacturer’s instructions and the laboratory’s validated workflow [32]. A log(score) ≥2.0 was considered reliable for species-level identification; isolates with scores of 1.70–1.99 were analyzed in duplicate.

Bacterial culture yielded pure growth of *Klebsiella pneumoniae* from each of the samples: liver, spleen, kidneys, lungs, myocardium, and long bone. No additional bacterial species were recovered from any of the submitted specimens. Species-level MALDI-TOF MS identification scores ranged from 2.21 to 2.48 across the six sites (Supplementary Table S2).

The in vitro antimicrobial susceptibility was assessed separately for each isolate using the Kirby–Bauer disk-diffusion method. Results were interpreted using EUCAST clinical breakpoints, version 16.0 [33], supplemented by CLSI M100, 36th edition [34], where applicable. All *K. pneumoniae* isolates exhibited the same reported disk-diffusion susceptibility profile. They were reported as resistant to ampicillin (intrinsic resistance), ampicillin–sulbactam, amoxicillin–clavulanic acid, piperacillin, and cefepime; susceptible to cefotaxime, amikacin, and tobramycin; and susceptible at increased exposure (I, EUCAST terminology) to ceftazidime and cefuroxime. Resistance was also reported for ciprofloxacin, ofloxacin, doxycycline, and trimethoprim; trimethoprim was tested alone, not in combination with sulfamethoxazole. Disk-diffusion screening also suggested presumptive imipenem non-susceptibility, which was not confirmed by minimum inhibitory concentration (MIC) determination or carbapenemase testing. Excluding intrinsic ampicillin resistance, the reported non-susceptibility involved seven antimicrobial categories: aminopenicillin/β-lactamase inhibitor combinations, antipseudomonal penicillins, fourth-generation cephalosporins, carbapenems, fluoroquinolones, tetracyclines, and folate-pathway inhibitors. The isolates were therefore described as multidrug-resistant (MDR) according to the criteria proposed by Magiorakos et al. [35], subject to confirmation of the underlying susceptibility interpretations. The antimicrobial disk contents and individual inhibition-zone diameters for all six isolates are provided in Supplementary Table S1.

Quality control was conducted using reference strains *Escherichia coli* ATCC 25922, *Staphylococcus aureus* ATCC 25923, and *Pseudomonas aeruginosa* ATCC 27853 to assess testing accuracy and repeatability. Clinical isolate results were recorded after verifying that reference-strain results fell within acceptable CLSI tolerance limits.

## 3. Discussion

This case combines clinical history, gross pathology, histopathology, bacteriology, and in vitro antimicrobial susceptibility testing in a geriatric companion chinchilla. The main pathological finding was severe bilateral chronic tubulointerstitial nephropathy with mineralization, whereas severe diffuse pulmonary congestion represented the most prominent acute lesion. Importantly, bacteria-associated inflammatory lesions were not identified in the histologically examined tissues, requiring cautious interpretation of the microbiological findings and precluding attribution of the terminal event to established disseminated septic disease.

The approximately six-month history of progressive weight loss and hyporexia, together with the chronic gross and histopathological renal changes, supports a long-standing renal disease process. Severe chronic renal disease may have contributed to the progressive clinical decline; however, a direct causal relationship between the renal lesions and the chronic weight loss and hyporexia cannot be established. Gastrointestinal tissues were not collected for histopathological examination; therefore, concurrent chronic gastrointestinal disease, including inflammatory or infiltrative disorders, could not be excluded. Chronic renal disease in companion mammals may have an insidious course, with nonspecific signs such as weight loss, reduced appetite, dehydration, and lethargy becoming apparent only in advanced disease [36]. Chinchillas, like other prey species, may also mask signs of illness until physiological reserve is substantially reduced [37,38].

The chronic gross renal abnormalities were supported histologically by tubular atrophy, interstitial expansion consistent with fibrosis, and glomerular and medullary mineralization, confirming advanced chronic renal injury. One kidney also contained a well-demarcated, indented cortical lesion that was grossly suggestive of a chronic infarct. Renal infarction may result from thromboembolic or other vascular events [39]; however, because the lesion was not specifically confirmed histologically in this case, its nature and pathogenesis could not be established, and a thromboembolic mechanism remains speculative. The cellular and nuclear debris observed within some glomeruli was a descriptive histological finding of uncertain significance and, in isolation, did not establish a distinct acute glomerular lesion.

The morphological pattern of advanced chronic tubulointerstitial nephropathy is not etiologically specific, and several processes could produce comparable end-stage renal changes. Although *K. pneumoniae* was recovered from both kidneys, intralesional bacterial colonies and suppurative or embolic nephritis were not identified in the examined renal sections; therefore, the findings do not support active bacterial nephritis at the time of death. Bacterial glomerulonephritis has previously been reported in a pet chinchilla [13], while endogenous oxalate nephrosis is another documented cause of renal disease in this species [40]. PAS, Masson–Goldner trichrome and von Kossa stains supported glomerular and tubular basement-membrane thickening, interstitial collagen deposition and mineralization, respectively.

As Congo red staining and polarized-light examination were not performed, amyloid deposition and intratubular oxalate could not be specifically evaluated. Age-related chronic nephropathy also remains a possible consideration, although its contribution cannot be established from the available findings. The renal lesions are therefore best reported morphologically as severe chronic tubulointerstitial nephropathy with mineralization rather than attributed to a specific etiology.

Mineralization was a prominent component of the renal lesions, with deeply basophilic mineral deposits identified within glomeruli and multifocally in the renal medulla. Renal mineralization may accompany chronic kidney disease and disturbances of mineral homeostasis, including alterations in calcium and phosphorus balance and secondary hyperparathyroidism [41], and mineralizing renal changes are recognized in exotic companion mammals [42]. In the present case, however, the underlying mechanism could not be determined from morphology alone. Serum calcium and phosphorus concentrations, parathyroid hormone measurements, and histological assessment of the parathyroid glands were not available, while von Kossa staining confirmed mineral deposition without establishing its underlying cause. Accordingly, mineralization is considered an important component of the chronic nephropathy in this case, while its pathogenesis remains undetermined.

Comparable gross lesions in domestic mammals can accompany chronic tubulointerstitial disease, chronic pyelonephritis, glomerular disease, fibrosis, and vascular injury [39]. In chinchillas, detailed reports of spontaneous renal disease remain limited, although bacterial glomerulonephritis associated with congenital diaphragmatic hernia [13] and renal infiltration by multicentric lymphoma [14] have been documented. Also, large retrospective mortality surveys have identified renal disease as an infrequent but recognized cause of death in chinchilla [10,11]. The present case therefore contributes additional morphological information on severe chronic renal disease in a geriatric companion chinchilla.

Chronic renal disease and systemic hypertension are closely associated in dogs and cats [43,44,45], but whether comparable cardiorenal mechanisms contributed in this chinchilla cannot be determined because blood pressure measurement, echocardiography, electrocardiography, serum biochemical data, and cardiac histopathology were unavailable. Accordingly, a cardiorenal relationship should not be inferred from the gross cardiac appearance alone.

Severe diffuse pulmonary congestion was one of the most prominent acute pathological findings and was considered clinically relevant in view of the progressive dyspnoea observed during the final 24 h. Grossly, the lungs were diffusely dark red, expanded, and poorly collapsed, raising suspicion of concurrent pulmonary oedema. Histologically, marked diffuse vascular congestion was confirmed; however, significant alveolar accumulation of oedematous/proteinaceous material, fibrin deposition, alveolar haemorrhage, fibrin microthrombi, or other evidence of acute alveolar injury was not identified in the examined sections. In addition, the absence of gross focal consolidation or abscess formation and of histological suppurative bronchopneumonia or intralesional bacterial colonies argues against established bacterial bronchopneumonia as the dominant pulmonary process. The mechanism underlying the severe pulmonary congestion could not be determined from the available post-mortem findings. Although progressive terminal dyspnoea was reported during the terminal period, pulmonary vascular congestion is a nonspecific finding that may also reflect agonal or hypostatic changes. Therefore, its contribution to the terminal event remains uncertain, and the available findings do not establish a definitive diagnosis of respiratory failure or its underlying mechanism.

Marked gastrointestinal gas accumulation occurred in the context of complete anorexia and absence of fecal output during the final 24–48 h. Gastrointestinal hypomotility is a common secondary response to pain, dehydration, anorexia, metabolic disturbance, and systemic illness in hindgut-fermenting small mammals [8,9]. In the absence of a gross obstructive lesion, hypomotility was considered a possible contributor to the observed gaseous distension; however, terminal or post-mortem gas accumulation could not be excluded. Because gastrointestinal tissues were not collected for histopathological examination, microscopic inflammatory, infiltrative, neoplastic, or other gastrointestinal disease could not be evaluated and may have contributed to the preceding chronic clinical signs.

A noteworthy result of the present investigation was the recovery of *Klebsiella pneumoniae* in pure cultures from six distinct samples, including the lungs, heart, liver, spleen, kidneys, and long bone. No additional bacterial species were isolated, and all isolates exhibited an identical in vitro antimicrobial susceptibility profile. Together, these findings are compatible with systemic bacterial dissemination or bacteremia. However, MALDI-TOF MS provides reliable species-level identification but does not establish strain-level relatedness. As molecular typing was not available, clonality of the isolates recovered from different organs could not be determined. In human post-mortem practice, monomicrobial recovery of a recognized opportunistic pathogen from multiple normally sterile sites in a body examined promptly and sampled with care is considered more suggestive of genuine ante-mortem invasion than mixed growth or recovery of typical contaminants [46,47]. Although direct extrapolation of these criteria to chinchillas requires caution, several features of the present case—including aseptic sample processing, pure growth of the same bacterial species from six sampled sites, prompt post-mortem examination, and rapid refrigerated transport—reduce concern for simple environmental or procedural contamination. They do not, however, distinguish definitively among ante-mortem bacteremia, perimortem bacterial dissemination, and early post-mortem translocation.

*Klebsiella pneumoniae*, a Gram-negative encapsulated bacterium belonging to the family Enterobacteriaceae, commonly colonizes the gastrointestinal and upper respiratory tracts of humans and animals but can act as an important opportunistic pathogen causing respiratory, urinary, hepatobiliary, wound, and systemic infections when host defenses are compromised [48,49,50]. Its pathogenicity is attributed to multiple virulence factors, including the polysaccharide capsule, lipopolysaccharide, fimbrial adhesins, siderophore-mediated iron acquisition systems, and biofilm-forming capacity, which facilitate immune evasion, persistence, tissue colonization, and systemic dissemination [48,49,50]. In addition to its virulence potential, *K. pneumoniae* has emerged as one of the most clinically significant multidrug-resistant bacterial pathogens in both human and veterinary medicine [51,52]. It has a substantial potential to acquire and disseminate mobile antimicrobial resistance determinants, making it a major contributor to the global spread of antimicrobial resistance within the One Health framework [51].

Reports of *K. pneumoniae* infection in chinchillas are scarce and document its association with disease in this species. A historical outbreak in a breeding colony was associated with anorexia, depression, respiratory distress, diarrhea, acute mortality, pulmonary lesions, and renal tubular injury [53]. In a retrospective study of 698 farmed chinchillas, *K. pneumoniae* was among bacterial agents recovered from fatal enteric and respiratory disease [10]. The present case differs from previously reported chinchilla infections because it involved a geriatric pet with substantial chronic pathology and no histological evidence of bacteria-associated inflammation in the examined tissues. As the time of death was not observed, the post-mortem interval before necropsy could have approached 6 h; therefore, post-mortem bacterial translocation remains a consideration despite aseptic sampling and rapid processing after discovery. When the microbiological and histopathological findings are considered together, the multiorgan recovery of *K. pneumoniae* does not establish the organism as the primary pathogen or definitive cause of death. The absence of characteristic bacteria-associated lesions does not completely exclude a peracute systemic bacterial process, particularly if dissemination occurred shortly before death; nevertheless, it limits the extent to which the microbiological findings can be assigned a causal role. Severe underlying chronic disease and terminal physiological compromise may also have facilitated bacterial translocation or dissemination. Accordingly, *K. pneumoniae* is best considered a possible opportunistic contributor to the terminal disease process or may have reflected perimortem bacterial dissemination. The portal of entry and timing of dissemination could not be established.

The antimicrobial treatment history provides little evidence for recent therapeutic selection of the MDR phenotype identified in this case. Antimicrobial exposure was limited to two short perioperative courses of enrofloxacin during early life, with no subsequent systemic antimicrobial treatment reported. Given the long interval between these treatments and death, a direct relationship between this remote exposure and the broad resistance phenotype observed post-mortem cannot be established. The source, timing of acquisition, and duration of carriage of the MDR *K. pneumoniae* therefore remain unknown. Nevertheless, the multidrug-resistant phenotype observed in this case is clinically relevant, especially because therapeutic options in exotic companion herbivores may be constrained not only by the antimicrobial resistance but also by species-specific adverse effects. Increasing antimicrobial resistance in *Klebsiella* spp. has been reported in companion animals [52], and resistant Gram-negative organisms are increasingly recognized in exotic pets [54]. Multidrug-resistant *K. pneumoniae* and other Enterobacterales have also been recovered from pet rabbits [55], indicating that antimicrobial resistance warrants attention across exotic companion herbivores. Furthermore, particular caution is required in chinchillas and other hystricomorph rodents because penicillins such as ampicillin and amoxicillin, lincosamides, erythromycin, and selected cephalosporins can disrupt the normal cecal microbiota and may precipitate severe dysbiosis and potentially fatal antibiotic-associated enterotoxemia [56,57,58,59]. Systemic aminoglycosides are not uniformly contraindicated in chinchillas but carry recognized nephrotoxic and ototoxic risks, which are especially relevant in an aged animal with severe chronic renal lesions [60]. Culture-guided, species-appropriate antimicrobial selection is therefore essential.

Disk-diffusion screening revealed an unusual reported phenotype characterized by cefepime resistance, presumptive imipenem non-susceptibility, and retained cefotaxime susceptibility. Confirmatory MIC determination, additional carbapenem testing, phenotypic carbapenemase assays, ESBL/AmpC assessment, and genotypic screening of resistance determinants were not performed; molecular typing to establish clonality of the six isolates was likewise unavailable. Consequently, reduced imipenem susceptibility is a presumptive screening observation rather than confirmation of carbapenem resistance mechanisms or carbapenemase production, and non-carbapenemase mechanisms cannot be excluded.

The recovery of MDR *K. pneumoniae* with presumptive imipenem non-susceptibility from a household pet also has potential One Health implications because close human–animal contact may create opportunities for exposure to resistant bacteria. However, neither owner exposure nor interspecies transmission was investigated in this case, and no zoonotic transmission can be inferred. The finding supports consideration of appropriate hygiene and prudent antimicrobial stewardship without establishing a specific source or direction of transmission.

Environmental heat exposure was temporally associated with the animal’s acute clinical deterioration. According to the owner, approximately 48 h before death, the chinchilla was exposed indoors for approximately 8 h to an ambient temperature estimated to have reached a maximum of 29 °C. The cage remained in the shade and drinking water was available ad libitum; however, no active ventilation or air conditioning was present. In light of the heat sensitivity of chinchillas outlined above, both the magnitude and duration of this exposure are potentially relevant. Experimental data indicate a risk of hyperthermia in *C. lanigera* at ambient temperatures around 30 °C [4], while heat-related illness and mortality have also been reported in captive chinchillas exposed to high environmental temperatures, particularly under unfavorable environmental conditions [10]. The estimated exposure to 29 °C in the present case therefore falls close to a temperature at which impaired thermoregulation has been demonstrated experimentally. Nevertheless, ambient temperature alone does not establish that the animal developed hyperthermia or heat stroke.

Heat stress can induce systemic physiological disturbances, including circulatory and inflammatory responses, although these mechanisms have not been specifically established in chinchillas [61,62]. In the present case, substantial pre-existing chronic disease may have reduced physiological reserve and increased susceptibility to an acute environmental stressor. The close temporal relationship between the heat exposure and subsequent rapid deterioration therefore supports a possible role for thermal stress as a precipitating factor in acute-on-chronic decompensation. However, its relative contribution to the terminal event cannot be determined retrospectively, and the available evidence is insufficient to establish heat stroke as a definitive diagnosis or cause of death.

Taken together, the findings do not support attribution of death to a single definitive cause. Severe bilateral chronic tubulointerstitial nephropathy with mineralization represented the principal underlying chronic disease process and may have reduced physiological reserve. Severe diffuse pulmonary congestion was observed alongside progressive terminal dyspnoea, however, the contribution of this nonspecific finding to the terminal event remains uncertain. Environmental heat exposure may have acted as a precipitating physiological stressor, although hyperthermia and heat stroke were not documented. The multiorgan recovery of MDR *K. pneumoniae* represents an important concurrent microbiological finding; however, in the absence of bacteria-associated inflammatory lesions in the histologically examined tissues, its causal contribution remains uncertain, and established disseminated septic disease cannot be demonstrated. Overall, the findings are most consistent with a multifactorial acute-on-chronic decompensation rather than a single established cause of death.

Several limitations should be considered when interpreting the findings of this case. No ante-mortem clinical or laboratory diagnostic investigations were performed, preventing assessment of renal function, systemic hypertension, and the functional significance of the cardiac changes identified post-mortem. In particular, serum biochemistry, urinalysis, blood pressure measurements, and diagnostic imaging were unavailable, and urinary parameters could not be compared with published reference values for clinically normal chinchillas [63]. Gastrointestinal tissues were not collected for histopathological examination; therefore, underlying gastrointestinal disease could not be excluded as a contributor to chronic weight loss and hyporexia, and the marked gaseous distension could not be attributed definitively to functional hypomotility rather than terminal or post-mortem changes. Interpretation of environmental heat exposure was limited by owner-estimated temperature and exposure duration, unavailable humidity measurements, and the absence of core body temperature measurements; consequently, hyperthermia was not documented, and heat stroke could not be confirmed. The animal was last known to be alive at approximately 03:30 and was found dead at approximately 09:00; post-mortem examination was initiated within 30 min of discovery, and the refrigerated organ samples reached the microbiology laboratory approximately 1 h after collection. Although necropsy and microbiological processing were performed promptly after discovery of the body, the time of death is unknown and post-mortem bacterial translocation could not be excluded. Molecular typing was not performed; therefore, despite the identical antimicrobial susceptibility profiles of the organ-derived isolates, their strain-level relatedness could not be established. Histopathological examination was available only for the kidneys, lungs, and liver, whereas the other microbiologically sampled sites were not examined histologically. Although PAS, Masson–Goldner trichrome, and von Kossa stains were performed to further characterize the renal lesions, Congo red staining and polarized-light examination were not undertaken; therefore, renal amyloidosis and intratubular oxalate crystals could not be formally excluded. In addition, the focal renal cortical lesion was grossly suggestive of a chronic infarct but was not specifically confirmed histologically; therefore, its nature and pathogenesis, including a possible thromboembolic mechanism, could not be established. Body weight at death and kidney weights were not recorded, precluding assessment of relative renal mass. Finally, as this report describes a single clinical case, the findings should not be generalized to the broader chinchilla population. Nevertheless, the case contributes to the limited clinicopathological knowledge of disease in geriatric companion chinchillas, particularly regarding severe chronic renal pathology and the interpretation of concurrent terminal abnormalities.

## 4. Conclusions

This case describes severe bilateral chronic tubulointerstitial nephropathy with glomerular and medullary mineralization, severe diffuse pulmonary congestion, marked gastrointestinal gaseous distension, and multiorgan isolation of multidrug-resistant *Klebsiella pneumoniae* in an 11-year-old companion chinchilla. Severe diffuse pulmonary congestion was observed together with progressive terminal dyspnoea; however, the contribution of this nonspecific finding to the terminal event remains uncertain, and its underlying mechanism could not be established. The absence of bacteria-associated inflammatory lesions in the histologically examined tissues warrants cautious interpretation of the microbiological findings and does not support established disseminated septic disease. *K. pneumoniae* may therefore have represented an opportunistic contributor to the terminal disease process or reflected perimortem bacterial dissemination. Environmental heat exposure may have acted as a precipitating stressor in an animal with severe underlying chronic disease and reduced physiological reserve; however, heat stroke could not be established. Overall, the findings are most consistent with multifactorial acute-on-chronic decompensation rather than a single definitive cause of death. This case expands the limited clinicopathological literature on geriatric companion chinchillas and highlights the value of integrating clinical history, gross pathology, histopathology, and microbiology when interpreting complex terminal disease in exotic companion mammals.

## Data Availability

Data supporting the findings of this case are available from the corresponding author upon reasonable request.

## Supplementary Materials

The following supporting information can be downloaded at: https://www.mdpi.com/article/10.3390/ani16213451/s1, Table S1: In vitro antimicrobial susceptibility profiles of Klebsiella pneumoniae isolates recovered from six anatomical sites in an 11-year-old chinchilla, determined by disk diffusion; Table S2: Culture and MALDI-TOF MS identification of isolates recovered at post-mortem examination.

## Abbreviations

The following abbreviations are used in this manuscript:

BALT	bronchus-associated lymphoid tissue
CLSI	Clinical and Laboratory Standards Institute
EUCAST	European Committee on Antimicrobial Susceptibility Testing
H&E	hematoxylin and eosin
MALDI-TOF MS	matrix-assisted laser desorption/ionization time-of-flight mass spectrometry
MDR	multidrug-resistant

## References

[1] Jenkins J.R. (2010). Diseases of Geriatric Guinea Pigs and Chinchillas. Vet. Clin. N. Am. Exot. Anim. Pract..

[2] Gorbunova V., Bozzella M.J., Seluanov A. (2008). Rodents for Comparative Aging Studies: From Mice to Beavers. Age.

[3] Darbyshire A., Osborne A., Jackson G., Fong D., Williams-Fritze M., Feldman E., Manuel C.A. (2023). The Chinchilla Consortium: Standardizing Health and Husbandry. Lab. Anim. Sci. Prof..

[4] Cortés A., Rosenmann M., Bozinovic F. (2000). Cost-Benefit Relationship in Thermoregulation of Chinchilla lanigera. Rev. Chil. Hist. Nat..

[5] Hsu C.C., Chan M.M., Wheler C.L., Fox J.G., Anderson L.C., Otto G.M., Pritchett-Corning K.R., Whary M.T. (2015). Biology and Diseases of Chinchillas. Laboratory Animal Medicine.

[6] Gilhofer E.M., Hebesberger D.V., Waiblinger S., Künzel F., Rouha-Mülleder C., Mariti C., Windschnurer I. (2024). Husbandry Conditions and Welfare State of Pet Chinchillas (*Chinchilla lanigera*) and Caretakers’ Perceptions of Stress and Emotional Closeness to Their Animals. Animals.

[7] Turner P.V., Brash M.L., Smith D.A. (2017). Chinchillas. Pathology of Small Mammal Pets.

[8] Mans C., Donnelly T.M., Quesenberry K.E., Orcutt C.J., Mans C., Carpenter J.W. (2020). Chinchillas. Ferrets, Rabbits, and Rodents: Clinical Medicine and Surgery.

[9] Reavill D. (2014). Pathology of the Exotic Companion Mammal Gastrointestinal System. Vet. Clin. N. Am. Exot. Anim. Pract..

[10] Martino P.E., Bautista E.L., Gimeno E.J., Stanchi N.O., Radman N.E. (2017). Fourteen-Year Status Report of Fatal Illnesses in Captive Chinchilla (*Chinchilla lanigera*). J. Appl. Anim. Res..

[11] Lucena R.B., Giaretta P.R., Tessele B., Fighera R.A., Kommers G.D., Irigoyen L.F., Barros C.S.L. (2012). Doenças de Chinchilas (*Chinchilla lanigera*). Pesqui. Vet. Bras..

[12] McCready J.E., Barboza T. (2024). Rodent Pediatrics. Vet. Clin. N. Am. Exot. Anim. Pract..

[13] Vetere A., Bertocchi M., Moggia E., Pelizzone I., Di Ianni F. (2021). Concomitant Congenital Diaphragmatic Hernia and Bilateral Bacterial Glomerulonephritis in a Pet Chinchilla (*Chinchilla lanigera*). BMC Vet. Res..

[14] Schlax K., Mélin M., Vieu S., Brune J., Vanel M., Risi E. (2026). Myocardial and Renal Lymphoma in a Long-Tailed Chinchilla (Chinchilla lanigera). Vet. Rec. Case Rep..

[15] Bousquet T., Friesen C., Hanak E. (2024). Atrial Septal Defect in a Chinchilla. J. Exot. Pet Med..

[16] Böttcher A., Wirth P., Eggert S. (2025). Granulosa Cell Tumor and Pyometra in a Fourteen-Year-Old Chinchilla (*Chinchilla lanigera*). J. Exot. Pet Med..

[17] McLaughlin A., Tripp C., Hostnik E., Travis A.-M., LaDouceur E. (2024). Diagnosis and Treatment of Metastatic Uterine Lymphoma in a Chinchilla (Chinchilla lanigera). Vet. Rec. Case Rep..

[18] Aymen J., Langlois I., Lanthier I. (2017). Diaphragmatic Hernia in a Pet Chinchilla (Chinchilla lanigera). Can. Vet. J..

[19] Ozawa S., Mans C., Miller J.L., Snyder C., Fazio C. (2019). Cleft Palate in a Chinchilla (Chinchilla lanigera). J. Exot. Pet Med..

[20] Long S., Qiao Y.J., Cao H., Chen Y., Shen Y. (2023). Case Report: Diagnosis and Treatment of a Chinchilla’s Old Radius and Ulna Fracture. Anim. Dis..

[21] Koizumi I. (2022). Diagnosis and Surgical Treatment of Colonic Obstructive Foreign Bodies in Chinchillas (Chinchilla lanigera): A Case Series (2017–2020). J. Vet. Med. Sci..

[22] Stephan F., Garnier P., Monge E., Manjon-Aspe A., Pignon C. (2026). Electrostimulation-Guided Radial, Ulnar, Median, and Musculocutaneous Nerve Block in a Chinchilla (Chinchilla lanigera) Undergoing Ulnar Intramedullary Pinning. Vet. Anaesth. Analg..

[23] Hirakawa Y., Sasaki H., Kawamoto E., Ishikawa H., Matsumoto T., Aoyama N., Kawasumi K., Amao H. (2010). Prevalence and Analysis of Pseudomonas aeruginosa in Chinchillas. BMC Vet. Res..

[24] Churria C.D.G., Vigo G.B., Origlia J., Campos J., Caffer M., Píscopo M., Loyola M.H., Petruccelli M., Pichel M. (2014). Diagnosis of an Outbreak of Salmonella Typhimurium in Chinchillas (Chinchilla lanigera) by Pulsed-Field Gel Electrophoresis. Rev. Argent. Microbiol..

[25] Berg C.C., Doss G.A., Mans C. (2019). Streptococcus equi Subspecies zooepidemicus Infection in a Pet Chinchilla (Chinchilla lanigera). J. Exot. Pet Med..

[26] Mitchell C.M., Johnson L.K., Crim M.J., Wiedmeyer C.E., Pugazhenthi U., Tousey S., Tollin D.J., Habenicht L.M., Fink M.K., Fong D.L. (2020). Diagnosis, Surveillance and Management of Streptococcus equi Subspecies zooepidemicus Infections in Chinchillas (Chinchilla lanigera). Comp. Med..

[27] Yarto-Jaramillo E., Hernández R.C., Rodríguez D., Çitaku I. (2026). First Report of Streptococcus canis-Associated Pneumonia and Pyothorax in a Pet Chinchilla (Chinchilla lanigera). J. Exot. Pet Med..

[28] Marques C., Belas A., Aboim C., Cavaco-Silva P., Trigueiro G., Gama L.T., Pomba C. (2019). Evidence of Sharing of Klebsiella pneumoniae Strains between Healthy Companion Animals and Cohabiting Humans. J. Clin. Microbiol..

[29] Menezes J., Frosini S.M., Weese S., Perreten V., Schwarz S., Amaral A.J., Loeffler A., Pomba C. (2024). Transmission dynamics of ESBL/AmpC and carbapenemase-producing Enterobacterales between companion animals and humans. Front Microbiol..

[30] Aziz S.J., Zeman-Pocrnich C.E. (2022). Tissue Processing. Methods Mol. Biol..

[31] Crăciun S., Novac C.Ş., Fiţ N.I., Bouari C.M., Bel L.V., Nadăş G.C. (2025). Bacterial Diversity in Pet Rabbits: Implications for Public Health, Zoonotic Risks, and Antimicrobial Resistance. Microorganisms.

[32] Crăciun S., Turcu M.C., Novac C.Ş., Fiţ N.I., Bouari C.M., Răpuntean S., Mihu M.L., Nadăş G.C. (2026). Pet Birds as Potential Reservoirs of Antimicrobial-Resistant Bacteria in Digestive and Respiratory Infections. Antibiotics.

[33] European Committee on Antimicrobial Susceptibility Testing (EUCAST) (2026). Breakpoint Tables for Interpretation of MICs and Zone Diameters.

[34] Clinical and Laboratory Standards Institute (CLSI) (2026). Performance Standards for Antimicrobial Susceptibility Testing.

[35] Magiorakos A.-P., Srinivasan A., Carey R.B., Carmeli Y., Falagas M.E., Giske C.G., Harbarth S., Hindler J.F., Kahlmeter G., Olsson-Liljequist B. (2012). Multidrug-Resistant, Extensively Drug-Resistant and Pandrug-Resistant Bacteria: An International Expert Proposal for Interim Standard Definitions for Acquired Resistance. Clin. Microbiol. Infect..

[36] Sparkes A.H., Caney S., Chalhoub S., Elliott J., Finch N., Gajanayake I., Langston C., Lefebvre H.P., White J., Quimby J. (2016). ISFM Consensus Guidelines on the Diagnosis and Management of Feline Chronic Kidney Disease. J. Feline Med. Surg..

[37] Divers S.J. (2012). Emergency Presentation of Exotic Mammal Herbivores. J. Exot. Pet Med..

[38] DeCubellis J. (2016). Common Emergencies in Rabbits, Guinea Pigs, and Chinchillas. Vet. Clin. N. Am. Exot. Anim. Pract..

[39] Cianciolo R.E., Mohr F.C., Maxie M.G. (2016). Urinary System. Jubb, Kennedy & Palmer’s Pathology of Domestic Animals.

[40] Goudas P., Lusis P. (1970). Case Report: Oxalate Nephrosis in Chinchilla (Chinchilla laniger). Can. Vet. J..

[41] Foster J.D. (2016). Update on Mineral and Bone Disorders in Chronic Kidney Disease. Vet. Clin. N. Am. Small Anim. Pract..

[42] Reavill D.R., Lennox A.M. (2020). Disease Overview of the Urinary Tract in Exotic Companion Mammals and Tips on Clinical Management. Vet. Clin. N. Am. Exot. Anim. Pract..

[43] Pouchelon J.L., Atkins C.E., Bussadori C., Oyama M.A., Vaden S.L., Bonagura J.D., Chetboul V., Cowgill L.D., Elliott J., Francey T. (2015). Cardiovascular–Renal Axis Disorders in the Domestic Dog and Cat: A Veterinary Consensus Statement. J. Small Anim. Pract..

[44] Brown S., Atkins C., Bagley R., Carr A., Cowgill L., Davidson M., Egner B., Elliott J., Henik R., Labato M. (2007). Guidelines for the Identification, Evaluation, and Management of Systemic Hypertension in Dogs and Cats. J. Vet. Intern. Med..

[45] International Renal Interest Society (IRIS) (2023). IRIS Guidelines for the Diagnosis, Staging and Management of Chronic Kidney Disease in Dogs and Cats. https://www.iris-kidney.com.

[46] Morris J.A., Harrison L.M., Partridge S.M. (2006). Postmortem Bacteriology: A Re-Evaluation. J. Clin. Pathol..

[47] Riedel S. (2014). The Value of Postmortem Microbiology Cultures. J. Clin. Microbiol..

[48] Paczosa M.K., Mecsas J. (2016). Klebsiella pneumoniae: Going on the Offense with a Strong Defense. Microbiol. Mol. Biol. Rev..

[49] Martin R.M., Bachman M.A. (2018). Colonization, Infection, and the Accessory Genome of Klebsiella pneumoniae. Front. Cell. Infect. Microbiol..

[50] Assoni L., Couto A.J.M., Vieira B., Milani B., Lima A.S., Converso T.R., Darrieux M. (2024). Animal Models of Klebsiella pneumoniae Mucosal Infections. Front. Microbiol..

[51] Wyres K.L., Holt K.E. (2018). Klebsiella pneumoniae as a Key Trafficker of Drug Resistance Genes from Environmental to Clinically Important Bacteria. Curr. Opin. Microbiol..

[52] Jiménez-Serrano M., Vidal A., Duran I., Seminati C., Darwich L. (2026). Evolution and Antimicrobial Resistance Profiles of Klebsiella spp. Infections in Companion Animals in the Iberian Peninsula. Antibiotics.

[53] Bartoszcze M., Matras J., Palec S., Roszkowski J., Wystup E. (1990). Klebsiella pneumoniae Infection in Chinchillas. Vet. Rec..

[54] Muñoz-Ibarra E., Molina-López R.A., Durán I., Garcias B., Martín M., Darwich L. (2022). Antimicrobial Resistance in Bacteria Isolated from Exotic Pets: The Situation in the Iberian Peninsula. Animals.

[55] Xin C., Hill F., Elsohaby I. (2024). Retrospective Analysis of Antimicrobial Resistance in Bacterial Pathogens from Pet Rabbits in Hong Kong, 2019–2022. J. Vet. Diagn. Investig..

[56] Ramirez H.E., Giguère S., Prescott J.F., Baggot J.D., Walker R.D., Dowling P.M. (2006). Antimicrobial Drug Use in Rodents, Rabbits, and Ferrets. Antimicrobial Therapy in Veterinary Medicine.

[57] Tamura Y. (2010). Current Approach to Rodents as Patients. J. Exot. Pet Med..

[58] Quesenberry K.E., Orcutt C.J., Mans C., Carpenter J.W. (2020). Ferrets, Rabbits, and Rodents: Clinical Medicine and Surgery.

[59] Wheler C.L., Dowling P.M., Dowling P.M., Prescott J.F., Baptiste K.E. (2024). Antimicrobial Therapy in Rabbits, Rodents, and Ferrets. Antimicrobial Therapy in Veterinary Medicine.

[60] Mercer M.A. Aminoglycosides Use in Animals. Merck Veterinary Manual. https://www.merckvetmanual.com/pharmacology/antibacterial-agents/aminoglycosides-use-in-animals.

[61] Bruchim Y., Horowitz M., Aroch I. (2017). Pathophysiology of Heatstroke in Dogs—Revisited. Temperature.

[62] Prates J.A.M. (2025). Heat Stress Effects on Animal Health and Performance in Monogastric Livestock: Physiological Responses, Molecular Mechanisms, and Management Interventions. Vet. Sci..

[63] Doss G.A., Mans C., Houseright R.A., Webb J.L. (2016). Urinalysis in Chinchillas (Chinchilla lanigera). J. Am. Vet. Med. Assoc..

