# Supplementary material for: Severe Chronic Tubulointerstitial Nephropathy in a Geriatric Pet Chinchilla (Chinchilla lanigera): A Case Report and Literature Review
Source: Animals (Basel). 2026 Nov 1;16(21):3451. doi: 10.3390/ani16213451 (PMC13761623; doi:10.3390/ani16213451)
Supplement: Supplementary file 1 [file animals-16-03451-s001.zip › animals-4611608-supplementary.pdf]

Table S1. In vitro antimicrobial susceptibility profiles of *Klebsiella pneumoniae* isolates recovered from six anatomical sites in an 11-year-old chinchilla, determined by disk diffusion.

Antimicrobial	Disk	Liver	Spleen	Kidney	Lung	Heart	Long bone	Interpretation	Interpretive criteria (mm)
Ampicillin (AMP)	10 µg	0	0	0	0	0	0	R (intrinsic)	Intrinsic resistance; not counted for acquired MDR
Ampicillin–sulbactam (SAM)	10/10 µg	0	0	0	0	0	0	R	EUCAST 16.0: S ≥14; R <14
Amoxicillin-clavulanic acid (AMC)	20/10 µg	0	0	0	0	0	0	R	EUCAST 16.0 (IV): S ≥19; R <19; ATU 19–20
Piperacillin (PIP)	30 µg	0	0	0	0	0	0	R	EUCAST 16.0: S ≥20; R <20
Cefuroxime (CXM)	30 µg	22.48	21.25	22.03	20.5	21.22	23.4	I	EUCAST 16.0 (IV): S ≥50; R <19; I 19–49
Cefotaxime (CTX)	5 µg	25.62	25.26	24.29	23.58	24.28	26.84	S	EUCAST 16.0: S ≥20; R <17; I 17–19
Ceftazidime (CAZ)	10 µg	20.81	19.87	20.59	20.47	19.79	21.12	I	EUCAST 16.0: S ≥22; R <19; I 19–21
Cefepime (FEP)	30 µg	0	0	0	0	0	0	R	EUCAST 16.0: S ≥27; R <24; I 24–26
Imipenem (IPM)	10 µg	0	0	0	0	0	0	R	EUCAST 16.0: S ≥22; R <19; I 19–21
Ciprofloxacin (CIP)	5 µg	15.32	15.45	14.64	14.89	15.47	16.48	R	EUCAST 16.0: S ≥25; R <22; ATU 22–24
Ofloxacin (OFX)	5 µg	12.64	13.84	12.98	13.87	13.85	13.15	R	EUCAST 16.0: S ≥24; R <22; I 22–23
Doxycycline (DOX)	30 µg	0	0	0	0	0	0	R	CLSI M100, 36th ed. (2026): S ≥14 mm; I 11–13 mm; R ≤10 mm
Trimethoprim (TMP)	5 µg	0	0	0	0	0	0	R	CLSI M100, 36th ed. (2026): S ≥16 mm; I 11–15 mm; R ≤10 mm
Amikacin (AMK)	30 µg	20.67	19.26	19.47	20.45	19.98	20.52	No acquired resistance detected	EUCAST 16.0 systemic bracketed threshold (18 mm); not clinical S

Tobramycin (TOB)	10 μg	18.15	17.45	17.21	18.05	19.32	17.78	No acquired resistance detected	EUCAST 16.0 systemic bracketed threshold (16 mm); not clinical S

Note: Values represent the original recorded inhibition-zone diameters (mm) for isolates recovered from six anatomical sites. Antimicrobial susceptibility was assessed separately for each isolate by disk diffusion. Interpretations were based on EUCAST clinical breakpoints, version 16.0 (2026), supplemented by CLSI M100, 36th edition (2026), for doxycycline and trimethoprim. EUCAST category I denotes susceptible, increased exposure. Bracketed EUCAST aminoglycoside thresholds indicate the absence of detected acquired resistance and should not be interpreted as evidence of clinical susceptibility. Imipenem resistance was indicated by disk diffusion but was not confirmed by MIC determination or carbapenemase testing. Intrinsic ampicillin resistance was excluded when evaluating acquired multidrug resistance.

Table S2. Culture and MALDI-TOF MS identification of isolates recovered at post-mortem examination.

**Specimen**	**Culture result**	**MALDI-TOF identification**	**MALDI-TOF score**	**Antimicrobial susceptibility profile**
**Liver**	Pure growth; no additional bacterial species recovered	*Klebsiella pneumoniae*	**2.47**	MDR; AMP-R, SAM-R, AMC-R, PIP-R, FEP-R, IPM-R, CIP-R, OFX-R, DOX-R, TMP-R; CTX-S, CAZ-I, CXM-I; AMK and TOB - no acquired resistance detected according to EUCAST bracketed systemic aminoglycoside thresholds
**Spleen**	Pure growth; no additional bacterial species recovered	*Klebsiella pneumoniae*	**2.32**	MDR; AMP-R, SAM-R, AMC-R, PIP-R, FEP-R, IPM-R, CIP-R, OFX-R, DOX-R, TMP-R; CTX-S, CAZ-I, CXM-I; AMK and TOB - no acquired resistance detected according to EUCAST bracketed systemic aminoglycoside thresholds
**Kidney**	Pure growth; no additional bacterial species recovered	*Klebsiella pneumoniae*	**2.48**	MDR; AMP-R, SAM-R, AMC-R, PIP-R, FEP-R, IPM-R, CIP-R, OFX-R, DOX-R, TMP-R; CTX-S, CAZ-I, CXM-I; AMK and TOB - no acquired resistance detected according to EUCAST bracketed systemic aminoglycoside thresholds
**Lung**	Pure growth; no additional bacterial species recovered	*Klebsiella pneumoniae*	**2.21**	MDR; AMP-R, SAM-R, AMC-R, PIP-R, FEP-R, IPM-R, CIP-R, OFX-R, DOX-R, TMP-R; CTX-S, CAZ-I, CXM-I;

				AMK and TOB - no acquired resistance detected according to EUCAST bracketed systemic aminoglycoside thresholds
**Heart**	Pure growth; no additional bacterial species recovered	*Klebsiella pneumoniae*	**2.42**	MDR; AMP-R, SAM-R, AMC-R, PIP-R, FEP-R, IPM-R, CIP-R, OFX-R, DOX-R, TMP-R; CTX-S, CAZ-I, CXM-I;<br>AMK and TOB - no acquired resistance detected according to EUCAST bracketed systemic aminoglycoside thresholds
**Long bone**	Pure growth; no additional bacterial species recovered	*Klebsiella pneumoniae*	**2.35**	MDR; AMP-R, SAM-R, AMC-R, PIP-R, FEP-R, IPM-R, CIP-R, OFX-R, DOX-R, TMP-R; CTX-S, CAZ-I, CXM-I;<br>AMK and TOB - no acquired resistance detected according to EUCAST bracketed systemic aminoglycoside thresholds

Note: AMP, ampicillin; SAM, ampicillin–sulbactam; PIP, piperacillin; FEP, cefepime; IPM, imipenem; CTX, cefotaxime; CAZ, ceftazidime; CXM, cefuroxime; CIP, ciprofloxacin; OFX, ofloxacin; DOX, doxycycline; TMP, trimethoprim; AMK, amikacin; TOB, tobramycin; R, resistant; S, susceptible; I, susceptible, increased exposure (EUCAST terminology); NS, non-susceptible; MDR, multidrug-resistant.
